# Biocidal Conditions in Low-Mars-Orbit Can Inactivate Bioburden on External Mars Spacecraft Surfaces and Dust Particles Within a Few Sols

**DOI:** 10.3390/microorganisms14051158

**Published:** 2026-05-20

**Authors:** Andrew C. Schuerger, Petra Schwendner, Lisa Guan, Jerami Mennella, Nicholas Heinz, Ioannis Mikellides, Brian G. Clement

**Affiliations:** 1 Department of Plant Pathology, University of Florida, Gainesville, FL 32611, USA; schuerg@ufl.edu (A.C.S.); science.schwendner@outlook.com (P.S.); 2 Jet Propulsion Laboratory, California Institute of Technology, Pasadena, CA 91109, USA; lisa.guan@jpl.nasa.gov (L.G.); jerami.mennella@jpl.nasa.gov (J.M.); nicholas.a.heinz@jpl.nasa.gov (N.H.); ioannis.g.mikellides@jpl.nasa.gov (I.M.)

**Keywords:** Mars Sample Return mission, planetary protection, Mars astrobiology, spacecraft bioburden

## Abstract

Mars Sample Return Program planning includes a series of spacecraft staged both on the Martian surface and in low-Mars-orbit (LMO). During the transfer of samples into orbit, external spacecraft surfaces might be exposed to Mars dust carried on the sample container exterior and possibly extant microbiota (if present). This study was designed to characterize the synergistic effects of LMO ultraviolet irradiation, vacuum, and solar heating on the survival of two UV-resistant and heat-tolerant bacteria, one yeast, and one fungus. The species tested were *Bacillus pumilus* SAFR-032 spores, *Geobacillus stearothermophilus* ATCC 12980 spores, *Naganishia onofrii* DBVPG 5303 cells, and *Aspergillus fumigatus* ISSFT-021-30 spores, respectively. Spores of *A. fumigatus* ISSFT-021-30 and *B. pumilus* were also exposed to LMO conditions with and without a Mojave Mars Simulant (MMS) dust layer. Based on the data, the time required to reach the desired Sterility Assurance Level (SAL; dose-defined to yield a −12 log reduction) was 2.0 h for *A. fumigatus* ISSFT-021-30 and 76.6 min for *B. pumilus* SAFR-032 if exposed directly to the solar UV beam under LMO conditions. With the MMS present, predicted times to reach one SAL were extended to 22 h and 1.72 h, respectively. Analysis of UV transmittance through cell stacks of up to 12 µm thick was performed for *A. fumigatus* ISSFT-021-30. Results indicated that ~4–5% of UVC photons can penetrate through 12 µm stacked aggregates of spores. These findings indicate that (1) the LMO environment can be used to attain the mandated levels of spacecraft surface bioburden reductions and (2) dust shielding and microbial aggregation attenuate UV irradiation, leading to extended orbital residence times to achieve mandated bioburden reductions.

## 1. Introduction

The National Aeronautical and Space Administration (NASA) and the European Space Agency (ESA) have been planning to bring samples back from Mars via a multi-mission sample return campaign [1] initiated by the Mars 2020 flight project that delivered the Perseverance Rover to Mars. Perseverance is designed to collect and cache samples of Mars regolith, rocks and atmosphere in advance of a sample return mission. The Mars Sample Return Program (MSRP), a joint NASA/ESA effort, is the organization responsible for the subsequent space flight missions that would return samples collected by Perseverance.

NASA and ESA require that missions to Mars implement planetary protection measures. Forward planetary protection measures are implemented in support of science investigations to prevent false positive findings of biological and organic materials from Earth [2,3,4]. Backward planetary protection measures are implemented during sample return missions to protect Earth’s biosphere from potentially harmful extraterrestrial material. Such measures are applied when a Restricted Earth Return is deemed necessary because the extraterrestrial target body has the potential to host life.

Sample returns from Mars have been classified as restricted returns and both NASA and ESA intend to implement backward planetary protection requirements to prevent potentially hazardous Mars material (PHMM) from reaching the Earth’s biosphere as part of MSRP [1,5]. Engineering requirements that would be levied on NASA flight hardware define PHMM as Mars particles ≥ 50 nm in size that have not been subjected to a sterilization process. Containment or sterilization of PHMM that may reach Earth as a result of the mission is a primary MSRP requirement. Vectors for PHMM include contact with the surface, which may be avoided by design, and exposure to aerosols derived from surface-exposed regolith and rocks, which requires intricate processes and hardware. Therefore, implementing aerosol exclusion or in-flight sterilization, either through the use of a dedicated spacecraft system (e.g., a heat or UV sterilization system) or through predictable exposure to environmental lethality factors, is a key mission architecture decision. The most recent MSRP planning architecture [6] would ensure that returning hardware is shielded from uncontained PHMM by aerosol exclusion. However, off-nominal scenarios, such as anomalies during surface operations or sample handling, in which uncontained PHMM may contaminate flight hardware and be released into free-flight once in space, must be assessed as contingency cases to ensure end-to-end mission safety.

The space environment near Mars imposes multiple lethality factors [7,8] that could serve as opportunities for environmentally induced sterilization of PHMM. In low-Mars-orbit (LMO), where hardware may reside for a period of time before being returned to Earth, the most destructive environmental factor is solar ultraviolet (UV) irradiation. However, the current planetary protection (PP) and Mars astrobiology literature lack empirical data on how the LMO environment may be biocidal to microbial life. Here we present findings from a series of experiments—under simulated LMO conditions—to investigate the potential biocidal nature of the LMO environment as part of understanding the fate of uncontained PHMM that reaches orbit as part of the proposed MSRP missions.

While the final architecture of the MSRP campaign is undergoing reassessment at the time of this study, the 2023 planning architecture for MSRP [1] included using a Sample Retrieval Lander (SRL) to collect cached samples on the surface and transfer the sample tubes to an Orbiting Sample (OS) container mounted on a two-stage rocket, the Mars Ascent Vehicle (MAV). Once the cached samples are retrieved, the OS will be closed with a particle-tight lid, and the MAV will launch the OS into LMO. In the 2025 planning architecture, the OS exterior would be protected from PHMM contamination through the implementation of a cleanroom-like enclosure for sample handling on the SRL [6]. This approach breaks the chain of contact to Earth upon leaving the Mars atmosphere and deploying the OS in LMO. The OS would remain in LMO for 30 to 70 sols before an Earth Return Orbiter (ERO) captured it inside a NASA-provided payload system, the Capture, Containment and Return System (CCRS). The CCRS would then stow the OS in a secondary containment vessel (the OS being the primary container) that resides in an entry vehicle for later delivery to Earth.

Should anomalies in surface or Mars launch operations occur, PHMM in aerosols could reach LMO uncontained, and two types of material would require mitigation: (1) Mars particles that remain attached to the OS or MAV surfaces upon reaching Mars orbit and (2) free-flying Mars particles that may have detached from the OS and upper stage of the MAV in orbit and reattach to the exterior of ERO. While in orbit, the orientation of the OS spin-axis is uncontrolled, and establishing that all surfaces receive sterilizing doses of solar UV would require long-term (~6 months) exposure to allow a ≥90 degree change in the sun-OS angle. In contrast to hardware-associated particles, free-flying particles would not self-shadow to a degree that prevents eventual sterilization by solar UV [9]. The rate at which sterilization occurs is of interest because once a free-flying particle adheres to the ERO spacecraft exterior, it has a non-zero probability of being on a surface that faces away from the sun and being released (emitted) on an Earth-return trajectory.

This study focuses on the solar UV light exposure required to sterilize potential Mars biology associated with surfaces and free-flying Mars particles, and assesses if the exposure time required is well within the free-flight durations for a particle reaching Earth via the OS and/or ERO. If solar illumination were shown to be sterilizing, other missions returning from Mars could meet backward planetary protection and engineering requirements by asserting that select Earth contamination vectors would be passively mitigated by solar exposure alone, instead of needing costly engineered mitigations. To assess the feasibility of asserting sterilization and establishing conservative rates, we explored doses of simulated solar UV applied under LMO conditions that are required to inactivate hardy microbes, both with and without light attenuation provided by Mars-like particles.

Experiments were designed to align with biomedical industry standards for sterilization definitions. Overkill sterilization is defined as a sterility assurance level (SAL) predicted to achieve 12-log microbial reductions (henceforth given as ‘−12 log’). The SAL is estimated by empirically establishing the biocidal dose required to achieve inactivation of 10^6^ spores/cells per replicate, and then doubling the timestep to yield a ‘predicted SAL’ at −12 log reduction [10,11]. SAL values are commonly used as threshold values in industry to demonstrate overkill sterilization processes for use with medical devices [12].

The primary goal here was to empirically demonstrate ≥6-log reductions for several UV-resistant microorganisms [11] under LMO conditions. Results would correspond to the overkill sterilization rates (i.e., SAL) called for in section 5.4.2.b of the NASA Technical Standard 8719.27 [13] for preventing harmful contamination of Earth’s biosphere with extraterrestrial material. Note that this is a 100-fold greater microbial reduction than proposed for functional sterilization in Schuerger et al. [14]. The term ‘sterilized’ is used here as a ‘functional’ term to represent the non-detection of survivors in the microbial assays described below, following the arguments of Schuerger et al. [14].

## 2. Methods

### 2.1. Microbiological Protocols

Strains were selected from Craven et al. [11] and Blachowicz et al. [15] based on their previously demonstrated UV resistance or heat tolerance. The following species were tested for their resistance to environmental conditions found in LMO (Section 2.3): *Aspergillus fumigatus* ISSFT-021-30 (UV-resistant fungus [15]) was sourced by the Jet Propulsion Lab (JPL), *Bacillus pumilus* SAFR-032 (UV- and heat-resistant spore-forming bacterium [11]) was sourced by JPL, *Geobacillus stearothermophilus* ATCC 12980, ATCC 12980 (high-heat resistance bacterium [11]) was purchased from Mesa Labs (Lakewood, CO, USA), and *Naganishia onofrii* DBVPG 5303 (high altitude UV-tolerant fungus [15]) was sourced by JPL.

Spores of *A. fumigatus* ISSFT-021-30 were grown on potato dextrose agar (PDA; Difco, Fisher Scientific, Waltham, MA, USA) at 22–24 °C for 28 d, harvested in sterile deionized water (SDIW), passed through multiple layers of sterile glass wool (i.e., autoclaved for 30 min), and purified by low-speed centrifugation. Endospores (henceforth spores) of *B. pumilus* SAFR-032 were grown in liquid media and concentrated as described previously [16,17]. Endospores (henceforth spores) of *G. stearothermophilus* ATCC 12980 were used as received from the vendor. Cells of *N. onofrii* DBVPG 5303 were grown on PDA at 15 °C for 7 d, harvested in SDIW, and used immediately, or no longer than 3 weeks before new cells were produced. Spores of *A. fumigatus* ISSFT-021-30 were used for up to 12 weeks after production with no loss of viability. Spores of *B. pumilus* SAFR-032 and *G. stearothermophilus* ATCC 12980 can persist for years without significant reduction in viability when stored at 4 °C in glass vials, and thus, were grown or purchased once and used for the duration of the experiments. All spores or cells were maintained at 4 °C prior to experimentation.

In order to expose spores or cells to simulated solar UVC (200–280 nm) and other LMO conditions, uniform monolayers of each species were required. All monolayers were created on uncoated aluminum 6061 coupons as described previously [18,19]. Aluminum coupons were purchased from Seton, Inc. under a special quote (#26924068; Branford, CT, USA). Original coupons were trimmed with tin snips to approx. dimensions of 17 mm × 26 mm and then heat-sterilized for 48 h at 130 °C.

Spores and cells were suspended in SDIW in which Triton X-100 was added at the rate of 1.0 CMC (i.e., the critical micelle concentration; CMC), above which micelles would form by aggregating surfactant molecules [19]). The 1.0 CMC Triton X-100 solutions (*v*/*v* 0.01375%) were filter-sterilized through 0.2 µm polypropylene filters (Puradisc 25, Fisher Scientific) prior to use. Coupons were handled aseptically throughout the described experiments.

Optical density (OD at 600 nm) of each spore or cell suspension was tailored for optimum deposition of each species. The goal was to approach 1.0 × 10^6^ spores or cells per coupon without crowding or multiple layers. As the ODs for each species were being optimized, images of the created monolayers were initially collected with a Keyence high-resolution video microscope (model VHX-7000, Keyence Corp., Osaka, Japan). The final ODs resulting in good quality monolayers for spore/cell solutions applied to coupons were 0.410, 0.285, 0.600, and 1.00, respectively, for *A. fumigatus* ISSFT-021-30, *B. pumilus* SAFR-032, *G. stearothermophilus* ATCC 12980, and *N. onofrii* DBVPG 5303.

Once the final protocol was developed for each species, monolayers were imaged with scanning electron microscopy (SEM) to confirm that monolayers of spores or cells dominated the coupons. Monolayers on the aluminum coupon were not fixed prior to imaging, but coupons were first mounted on standard SEM stubs, sputter-coated with a gold-palladium electron conductive layer, and imaged with a Hitachi SU-5000 Schottky Field-Emission SEM (Hitachi, Dallas, TX, USA).

In all cases, a subtle ‘coffee ring’ effect [20,21] was observed for the edges of all microbial monolayers (Figure S1). Imaging the monolayers with the Keyence (Figure S2) and SEM (Figures S3 and S4) microscopes indicated that the coffee rings typically had no more than 1–2 layers of spores or cells. Thus, the vast majority of the applied spores or cells yielded monolayers across most of the doped regions of the coupons, with periodic small zones or sections of the coffee rings that exhibited 1–2 layers of stacked spores. The protocols described by Schuerger [18] and Schuerger and Headrick [19], and used herein, generated the best monolayers with the existing technologies available.

Once the quality of the dried coupons was confirmed, the species/coupons were exposed to LMO conditions for various time steps (see Section 2.3) using the Planetary Atmosphere Chamber (PAC) System (see Section 2.2). After exposure, coupons were removed from the Mars chamber and assayed by one of three possible assay protocols. Three experiments were conducted in series to characterize the effects of simulated solar UV irradiation, temperature, and low pressure in LMO on microbial survival. Each experiment had a unique design and microbial assay protocol (see Section 2.3).

### 2.2. Planetary Atmospheric Chamber (PAC) System

LMO simulations were conducted in the PAC located at the Space Life Sciences Lab adjacent to the Kennedy Space Center, Cape Canaveral, FL, USA. The PAC was originally described by Schuerger et al. in 2008 and 2011 [22,23]. In 2021, a turbo pump system (Twis Torr 304, Agilent Technologies, Santa Clara, CA, USA) was added to the PAC, allowing for the simulation of interplanetary conditions down to 1 × 10^−5^ mbar. Coupons were exposed to UV irradiation when the PAC internal pressure was between 1 and 5 × 10^−5^ mbar.

**Figure 1:**
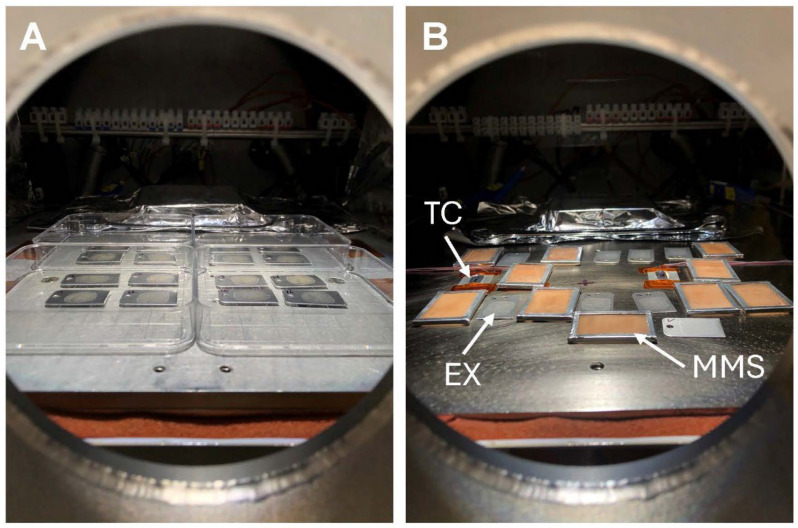
Coupon arrangements within the Planetary Atmosphere Chamber (PAC) during low-Mars-orbit (LMO) simulations. (**A**) The coupons for Exp-1 and Exp-2 were held inside sterile polystyrene petri dishes and placed in the central part of the UV beam within the PAC. (**B**) To enhance thermal cooling of coupons in Exp-3, coupons were placed directly on the upper surface of the stainless-steel liquid nitrogen (LN2) control plate. Exp-3 compared survival rates for two species on UV-exposed aluminum coupons and doped coupons covered by the Mojave Mars Simulant (MMS) filters (see text). The coupon temperatures were estimated by comparing four thermocouples (TC) placed left-to-right within the UV beam.

The solar UV (200–400 nm), visible (VIS; 400–700 nm), and near infrared (NIR; 700–1100 nm) spectral bands were created by a xenon arc lamp (model 6269, Newport, Corp., Mountain View, CA, USA; see Schuerger et al. [17,22]). Spectral bands were calibrated with an Optronics OL-754 UV-VIS-NIR spectroradiometer (model OL754, Optronics Lab, Orlando, FL, USA) and a hand-held UVC (200–280 nm) sensor (model IL1400A, International Light, Inc., Newburyport, MA, USA). The Mars UVC flux in LMO was set to 3.0 W/m^2^ (i.e., 10.8 kJ/m^2^/h). The uniformity of the UV beam at the locations of the coupons (Figure 1) was ±0.5 W/m^2^.

The UVC value was an average flux in LMO at 1.542 astronomical unit (AU; i.e., solar longitude [*Ls*] 180) given by two Mars UV models (Moores et al. [24], UVC = 2.89 W/m^2^; Vincente-Retortillo et al. [25], UVC = 3.10 W/m^2^). The LMO UVC dose was estimated to be 86.4 kJ/m^2^/sol for 8 h, which was treated as a 1-sol LMO simulation. This convention would hold for space flight hardware in LMO, which rotates around an axis perpendicular to the sun at a rate ≥ 1 rotation per orbit, and does not experience extended eclipses on each orbit. Surfaces on hardware orbiting under different conditions would experience a different UVC dose rate.

Temperature (15 or 37 °C; see Section 2.3) was controlled within the PAC by either a liquid nitrogen (LN2) thermal control plate (model TP1265, Sigma Systems, Corp., San Diego, CA, USA) or an in-house cryogenic liquid thermal cold plate that used a cryogenic fluid (Kryo-30, Lauda, Brinkman Instruments, Inc., Westbury, NY, USA) and a Lauda RE120 water chiller (Brinkman Instruments, Inc.). Hardware surfaces would see a greater range of temperatures in LMO depending on orbital parameters and thermal properties. The two temperatures tested in these experiments represent cases in which potentially confounding biochemical activity, such as photo repair or reactive oxygen species scavenging, might occur. Experiments at the temperatures tested here allowed conservatism in dose–response estimates if temperature-dependent biochemical activity were present at one or both temperatures.

### 2.3. Experimental Design and Microbial Assay Protocols

**Experiment 1.** The first experiment (Exp-1) was designed to characterize the UV-resistance of four microbial species exposed to 1-sol simulations (i.e., 8 h of UVC irradiation) under analog LMO conditions of (a) 15 or 37 °C, (b) 3.0 W m^−2^ UVC (200–280 nm), and (c) low pressures between 1 and 5 × 10^−5^ mbar. The average cell densities per coupon were estimated for *B. pumilus* SAFR-032 (1.88 × 10^6^), *G. stearothermophilus* ATCC 12980 (9.15 × 10^5^), *A. fumigatus* ISSFT-021-30 (1.67 × 10^5^), and *N. onofrii* DBVPG 5303 (2.38 × 10^4^). The lower cell densities for *A. fumigatus* ISSFT-021-30 and *N. onofrii* DBVPG 5303 were required due to the larger spore sizes of both species (approx. 3 µm in diameter), requiring lower cell densities per coupon to reduce stacking.

Doped coupons of each species were exposed to the LMO conditions in separate assays. Aluminum coupons doped with spores or cells were placed into sterile 100 mm × 100 mm square polystyrene petri dishes and clustered around the center of the UV beam within the PAC (Figure 1A). The coupons were crowded around the center of the UV beam, avoiding a small hotspot at the center of the beam.

Cohorts of doped coupons for each species were exposed to the following conditions: (1) time step zero controls (T0) which were doped coupons assayed immediately after the monolayers were dry (*n* = 4 per assay), (2) lab controls maintained in the biosafety hood at 22–24 °C for the duration of each assay (*n* = 4 per assay), (3) internal PAC controls that were placed within the PAC system and exposed to LMO conditions except shielded from UV irradiation (*n* = 4 per assay), and (4) PAC-LMO exposure coupons that received all three LMO conditions of low pressure, select temperatures (15 or 37 °C), and simulated solar UVC irradiation (*n* = 16 per assay). Assays for all species were conducted twice, yielding *n* = 8 for treatments #1–3 and *n* = 32 for the full LMO simulations.

The PAC system was operated as follows: (1) cohorts of coupons in sterile petri dishes were placed on the cryogenic cold plate and clustered towards the central 2/3 of the UV beam, (2) the PAC door was closed and the internal void space equilibrated to the LMO conditions listed above for 18 h, (3) the xenon arc UV beam was turned on for 8 h (i.e., a 1-sol simulation), (4) the PAC system was then warmed or cooled to 24 °C, (5) vented to 1015 mbar with ultra-high purity nitrogen gas, and (6) the coupons were removed and assayed.

After LMO exposures, coupons were assayed with a ‘standard’ Most Probable Number (MPN) assay described previously [18,19]. In brief, LMO-exposed coupons were transferred to a UV-sterilized biosafety hood (model NU-440-600, NuAire, Inc., Plymouth, MN, USA), and the monolayers were covered with 125 µL of a 10% polyvinyl alcohol (PVA) solution. The PVA layers were dried for 3–4 h within the NuAire biosafety hood in open petri dishes. The dried PVA films (i.e., stiff but also flexible) were then peeled off the coupons, placed in 10 mL of SDIW, vigorously shaken for 30 s, serial diluted to 10^−6^, and then 20 µL of each dilution dispersed into 16 wells of separate 96-well microtiter plates with 180 µL of various liquid media (i.e., six dilutions transferred to each 96-well microtiter plate). Trypticase soy broth (TSB) was used for standard MPN assays for *B. pumilus* SAFR-032 and *G. stearothermophilus* ATCC 12980. Potato dextrose broth (PDB) was used for standard MPN assays for *A. fumigatus* ISSFT-021-30. Spores of *N. onofrii* DBVPG 5303 from each dilution were streak plated on PDA and incubated at 23 °C for 7 days.

In treatments in which extremely low numbers of surviving spores were anticipated (e.g., LMO-UV exposures), an ‘extended’ MPN assay was used in which 20 µL per well from each PVA stock solution (i.e., 10^0^ dilution) was transferred to all 96 wells of the microtiter plates (i.e., a total of 1.92 mL from the stock PVA extraction fluid was dispensed per coupon). The combination of standard- and extended-MPN assays ensured the most accurate and reliable quantification of survivors per coupon. For *N. onofrii* DBVPG 5303, the stock suspension of spores was also plated on PDA and incubated as described above.

**Experiment 2.** A second experiment was developed in which doped coupons were exposed to LMO conditions at 37 °C for up to 12-sol simulations. Exp-2 was conducted with spores of *B. pumilus* SAFR-032 and *A. fumigatus* ISSFT-021-30 created as described above for Exp-1. Coupons were doped with spore densities in which individual coupons for *B. pumilus* SAFR-032 contained approx. 1.96 × 10^6^ spores/coupon. However, spores crowding remained an issue for *A. fumigatus* ISSFT-021-30, and thus, three coupons for *A. fumigatus* ISSFT-021-30 were pooled for individual replicates to achieve the target of exposing 10^6^ spores per replicate. The combined three coupons (i.e., 1 replicate) for *A. fumigatus* ISSFT-021-30 had an average spore density of 1.25 × 10^6^ spores per replicate.

Coupons doped with *B. pumilus* SAFR-032 spores were exposed to 1-h, 1-sol, 3-sol, or 12-sol LMO simulations (*n* = 32 replicates per LMO time step). Coupons doped with *A. fumigatus* ISSFT-021-30 spores were exposed to 3-, 6-, or 12-sol simulations (*n* = 24 replicates per LMO time step). Coupons were configured within the PAC system in a similar manner as described for Exp-1; namely, using the square petri dishes with coupons clustered towards the center 2/3 of the UV beam, as shown in Figure 1A. The PAC was operated as described for Exp-1. The temperature during UV exposures for Exp-2 was maintained at 15 °C for all assays.

Individual replicates were assayed by transferring the appropriate number of coupons equal to 1 replicate into separate 50 cc polypropylene conical tubes containing 15 mL of PDB or TSB for *A. fumigatus* ISSFT-021-30 (3 coupons per replicate) or *B. pumilus* SAFR-032 (1 coupon per replicate), respectively. Cultures were incubated at 30 °C for 7 or 3 days, respectively. Each 50-cc conical tube was rated as either [−] or [+] based on a visual assessment of turbidity indicating microbial growth. The data were plotted as the percentages of [+] tubes that exhibited growth per species at each time step.

**Experiment 3.** A third experiment was developed to characterize dust shielding on the biocidal effects of LMO-UV conditions. Filters were developed at JPL that sandwiched thin layers of a Mojave Mars Simulant (MMS [26,27]) with an adhesive matrix (Room Temperature Vulcanized (RTV) silicone (RTV108 Translucent Adhesive, Momentive, Inc., Waterford, NY, USA) between fused silica glass windows (1 mm UVFS Broadband Precision Window, Uncoated, Thorlabs, Inc., Newton, NJ, USA). The MMS filters were sealed at edges with an aluminum-adhesive tape (ACE, Oak Brook, IL, USA). MMS filters were heat-sterilized at 130 °C for 48–72 h prior to use. Ultraviolet scans of all MMS filters were obtained at the beginning and end of Exp-3 using a UV/VIS/NIR Spectrometer Lambda 1050 (Perkin Elmer, Shelton, CT, USA) to assess any changes resulting from handling, sterilization, or UV exposure during the experiment.

**Figure 2:**
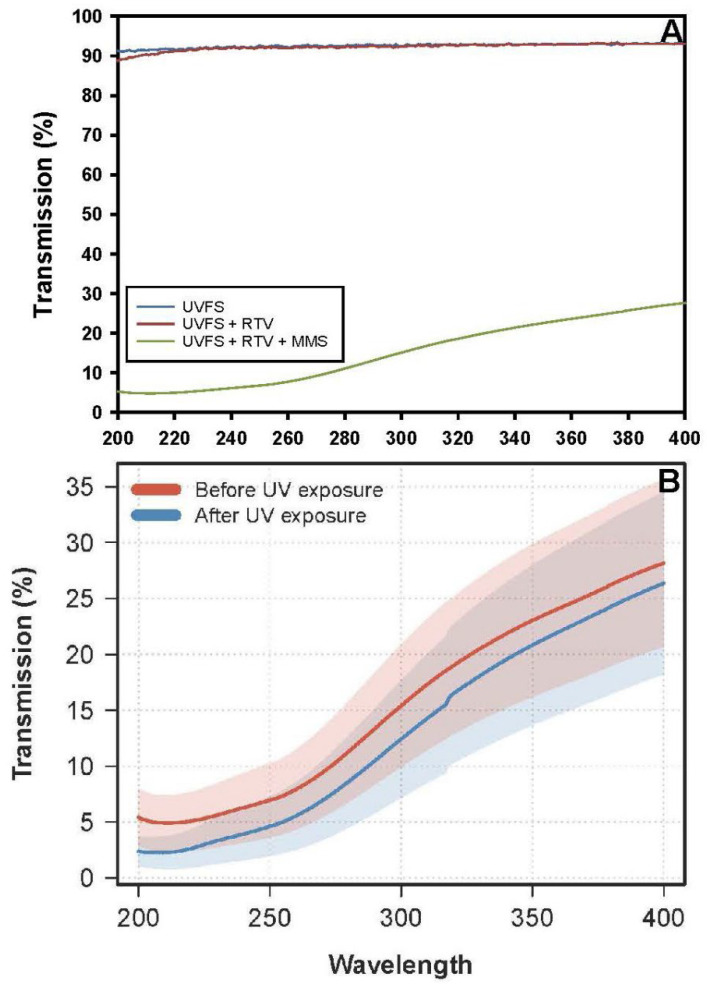
Ultraviolet (UV) transmittance through the Mojave Mars Simulant (MMS) filters (see text) used to attenuate UV irradiation in Exp-3. (**A**) The UV transmittance of the fused-silica (FS) glass was approx. 90–92% of the unattenuated beam. The use of a thin layer of RTV (i.e., used to hold MMS dust in place) had nearly identical UV transmittance compared to the FS-glass between 220 and 400 nm. The addition of the MMS dust to the RTV in a double FS-glass ‘sandwich’ significantly increased UV attenuation by 90–98%. (**B**) Ultraviolet (UV) transmission of the MMS filters measured for pre- and post-LMO simulations. The average UVC (200–280 nm) transmission of 26 MMS filters was lower post-LMO simulations by approx. 50% at 200 nm, 27% at 250 nm, and 22% at 280 nm. Longer UVB (280–320 nm) and UVA (320–400 nm) spectral ranges exhibited reduced levels of UV degradation of the MMS filters, with the reduction measured as 9% at 400 nm. Shaded areas in Figure 2B are 95% CI ranges.

At the start of Exp-3, the fused silica glass and the RTV adhesive matrix exhibited approx. 90% transmittance (T) of the UV beam (Figure 2A). When the MMS dust was added to the RTV matrix within the filters, UV attenuation was significantly increased and yielded an overall average of 5% T at 200–240 nm, 10% at 240–280 nm (both bands equal to the UVC band between 200 and 280 nm), 12% T for UVB (280–320 nm), and 20% T for UVA (320–400 nm).

The MMS glass filters were scanned for UV transmittance before and after all assays were conducted in Exp-3. The average UVC transmittance of all 26 MMS filters was lower post-LMO simulations by approx. 50% at 200 nm, 27% at 250 nm, and 22% at 280 nm (Figure 2B). Longer UVB (280–320 nm) and UVA (320–400 nm) irradiation exhibited reduced levels of UV degradation of 20% at 300 nm and 9% at 400 nm. The results suggest that the heat-sterilization cycles and/or the PAC UV irradiation during the LMO simulations may have aged the RTV silicone sealant used to create the MMS filters.

Spores were applied to aluminum coupons as described for Exp-1. The spore densities of individual coupons for Exp-3 were 5.69 × 10^5^ and 1.79 × 10^6^ for *A. fumigatus* ISSFT-021-30 and *B. pumilus* SAFR-032, respectively. Replicates for Exp-3 were comprised of individual coupons for both species.

A new configuration of the coupons within the PAC system was used for Exp-3 (Figure 1B). In the new configuration, four thermocouples (TC; model SA1-T, Omega, Inc., Stamford, CT, USA) were placed in a line directly on the stainless-steel surface of the LN2 control plate, and experimental coupons were randomly arranged around the TC wires. Two cohorts of coupons were laid out as follows: (1) eight coupons per assay were randomly arranged around the TCs and left exposed to the UV irradiation, and (2) eight coupons per assay were randomly arranged around the TCs but were covered by MMS filters (see Figure 1B). Each species and time step was conducted twice (*n* = 16). Removing the polystyrene square petri dishes and placing the coupons directly onto the cold plate enhanced the thermal heat exchange between the LN2 cold plate and the aluminum coupons. Exp-3 was conducted at 15 °C.

After coupons were placed on the LN2 cold plate surface, the PAC system was equilibrated to LMO conditions listed above in Exp-1. However, the PAC simulations were conducted for approx. 4 h at 1 to 5 × 10^−5^ mbar, in which UV-exposures were timed to begin at the 2 h mark. Coupons were exposed to the simulated solar UV beam for 0, 1, 5, 10, or 30 min. After a total elapsed time of 6 h in the PAC system, the chamber was vented to 1015 mbar, warmed to 24 °C, and the coupons assayed using the standard MPN assay.

### 2.4. Imaging and UV Transmission Through Microbial Monolayers

Additional coupons of *A. fumigatus* ISSFT-021-30 were prepared, inactivated via PAC UV exposure (see below), and sent to JPL for confocal microscopy and profilometry. At JPL, the coupons were examined for the structures of cell monolayers that had significant clumping and could therefore affect results if the clumps resulted in UV shielding. Ultraviolet inactivation of spores was achieved by exposing dried monolayers to 6 h of the PAC UV-VIS-NIR beam with the UVC flux adjusted to be 3.0 W/m^2^ [17,22,23].

To quantify the potential shielding effect of multiple cell layers, a cell suspension of *A. fumigatus* ISSFT-021-30 in SDIW was prepared at JPL and deposited onto UV-fused silica slides (Thorlabs, Newton, NJ, USA) via liquid deposition [18,19]. After drying, the slides were scanned using confocal microscopy to find areas of the monolayers with varying levels of cell stacking. Ultraviolet transmission was measured through cell stacks measuring approx. 2–3, 4–5, or 10–12 µm thick.

Optical measurements of spore aggregates were conducted using a CRAIC 2030PV PRO Microspectrophotometer (CRAIC Technologies, San Dimas, CA, USA), which combines a UV-VIS-NIR range optical microscope with a UV-VIS-NIR range spectrophotometer. In transmission micro-spectroscopy mode, light from the lamp housing is focused onto the sample on the microscope slide. The light transmitted through the sample was collected by the objective and directed to the entrance aperture of the spectrophotometer. An aperture size of 6 µm was used for the analysis.

Micro three-dimensional (3D) imaging was performed using a Keyence VK-X3100 Laser Scanning Confocal Microscope (LSCM) (Keyence Corporation of America, Itasca, IL, USA). The LSCM is an optical imaging technique that uses a focused laser beam scanned across a sample and a spatial pinhole to exclude out-of-focus light, allowing for high-resolution 2D and 3D images. This approach provides detailed information on surface topography, structure, and spatial distribution of microbial cells or particles on the sample.

### 2.5. Statistics

Non-detect values in Exp-1 were replaced by random numbers from a uniform (0,1) distribution, meaning that any number between zero and one was equally likely to occur. Data were normalized by dividing each MPN value after an LMO exposure (N) by the appropriate control (N_0_). Log10-transformed data were then analyzed using mixed-models methodology as implemented in PROC GLIMMIX (SAS/STAT^TM^ 15.2; SAS Institute, Cary, NC, USA). Heterogeneity of treatment variances was identified and normalized using graphical means as suggested by Kozak and Piepho [28] and dealt with by grouping residual variances into homogeneous groups using the ‘Group = option’ in the ‘Random Residual statement’ within PROC GLIMMIX. Least-squares for PAC Treatment versus Temperature interactions were calculated using the LSmeans statements in PROC GLIMMIX. Simple effect comparisons among treatments within temperature were conducted using the ‘pdiff’ option within the LSmeans statement without any adjustment for multiple comparisons, following arguments by Saville [29].

Data in Exp-3 were analyzed with PROC REG to generate linear models. A spline point for UV-exposed coupons for *B. pumilus* SAFR-032 (i.e., not covered by MMS filters) was estimated by solving the paired equations for the 1st and 2nd phases of the biocidal plots using the online app, www.wolframalpha.com (accessed on 5 April 2026), in ‘Approximate Mode’ (see Schuerger et al. [30]).

## 3. Results

### 3.1. Experiment 1: Effects of LMO Conditions on Microbial Survival for 1-Sol Simulations

Exp-1 was constrained to 1-sol LMO simulations (i.e., 48 h of vacuum and 8 h for UV irradiation) as a first-order test to characterize the biocidal nature of the LMO environment. The 1-sol LMO simulations were performed at two different temperatures (i.e., 15 °C and 37 °C) and pressures ≤ 5.0 × 10^−5^ mbar to determine whether the lethal effects under LMO conditions were attributable to UV exposure alone or to the combined influence of UV and other stressors.

**Figure 3:**
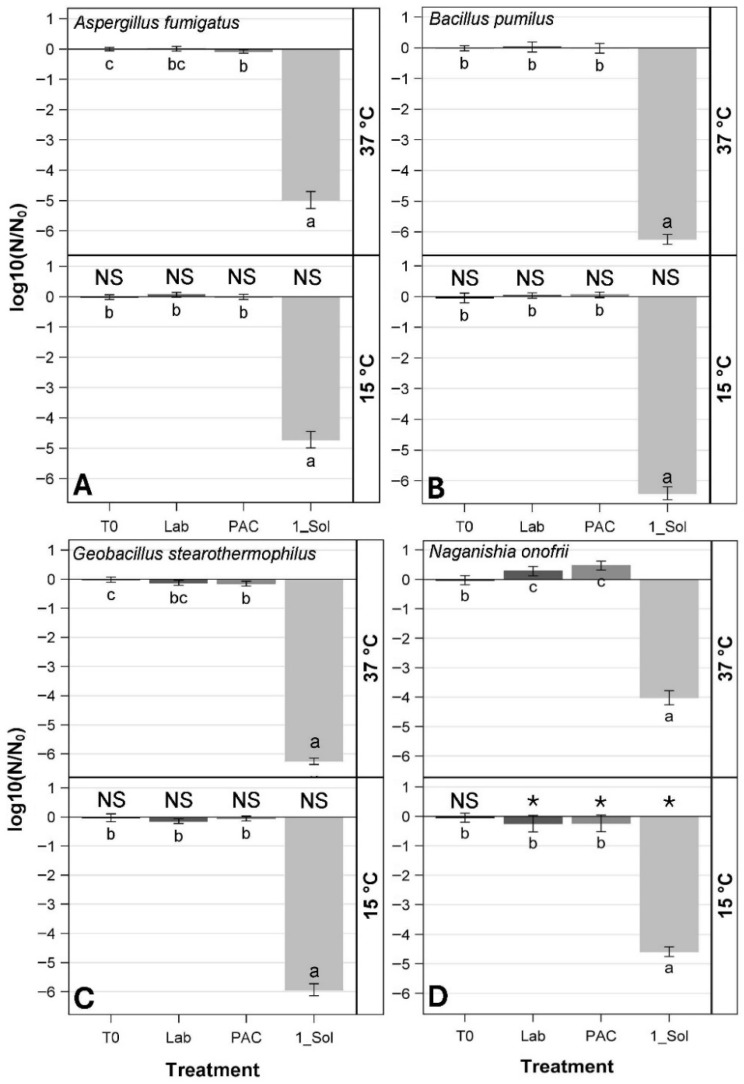
Log10 reductions in spores or cells for four species of UV-resistant microorganisms exposed to 1-sol simulations under low-Mars-orbit (LMO) conditions (Exp-1). Greater than −6 log reductions were observed for *Bacillus pumilus* SAFR-032 (**B**) and *Geobacillus stearothermophilus* ATCC 12980 (**C**), but not for *Aspergillus fumigatus* ISSFT-021-30 (**A**) and *Naganishia onofrii* DBVPG 5303 (**D**). Letters indicate significant differences between means (i.e., per species) based on ANOVA and protected least-squares means tests (*p* ≤ 0.05; *n* = 8 for T0, Lab, and PAC controls; *n* = 32 for all UV-exposed coupons). Comparisons between temperatures for each treatment are indicated by an asterisk (*) (*t*-tests; *p* ≤ 0.05). Abbreviations: T0 = time-zero controls, Lab = lab benchtop controls; PAC = internal Planetary Atmosphere Chamber controls (i.e., no UV); 1_Sol= PAC UV-exposed to a 1-sol LMO simulation; NS = not significant *t*-tests between temperatures. The UVC dose for the 1-sol LMO simulations was 3.0 W/m^2^ (10.8 kJ m^−2^h^−1^). Error bars are 95% CI ranges for treatment means.

At 15 °C, comparisons among the three controls (i.e., T0, Lab, and PAC controls) exhibited no significant differences for all four species (*p* > 0.05) (Figure 3). In contrast, at 37 °C, a few subtle differences were observed for *A. fumigatus* ISSFT-021-30 (Figure 3A), *G. stearothermophilus* ATCC 12980 (Figure 3C), and *N. onofrii* DBVPG 5303 (Figure 3D; *p* ≤ 0.05). The T0, Lab, and PAC controls were all similar for both 15 and 37 °C for *B. subtilis* spores (Figure 3B; *p* > 0.05). In addition, temperature comparisons were investigated for each individual species between 15 °C and 37 °C, in which most comparisons exhibited no significant differences (i.e., NS abbreviations in each Figure 3 sub-plot; *p* > 0.05). An exception was noted for temperature effects on *N. onofrii* DBVPG 5303 (Figure 3D) in which there was an observed difference between LMO simulations (*p* ≤ 0.05) 15 °C versus 37 °C (i.e., NS at *p* > 0.05), but significant comparisons (i.e., asterisks) were also observed for the Lab and PAC controls, indicating that the observed differences are likely due to batch variations rather than temperature effects. Results generally indicate that the LMO low-pressure and temperature conditions had little to no effect on microbial survival for all four species, and that the significant reductions in viability can be attributed to the applied UV dose.

*B. pumilus* SAFR-032 and *G. stearothermophilus* ATCC 12980-doped coupons averaged 1.88 × 10^6^ and 9.15 × 10^5^ spores deposited per coupon, respectively (Table 1). When exposed to 1-sol simulations, these two species exhibited bioburden losses of approx. −6 logs (Figure 3B and Figure 3C, respectively). However, spores of *A. fumigatus* ISSFT-021-30 and *N. onofrii* DBVPG 5303 were approx. three times larger than the two bacteria, and thus, the populations per coupon had to be reduced to limit unwanted stacking of spores. Spore stacking has been shown previously to protect underlying spores from UVC irradiation [18,19,30]. The T0 populations were 1.67 × 10^5^ and 2.38 × 10^4^ spores per coupon for *A. fumigatus* ISSFT-021-30 and *N. onofrii* DBVPG 5303, respectively (Table 1). Because the starting populations for *A. fumigatus* ISSFT-021 and *N. onofrii* DBVPG 5303 were lower than the two bacteria, the levels of bioburden reductions were constrained by the protocol, yielding approx. −5 logs for *A. fumigatus* ISSFT-021 and approx. −4.5 logs for *N. onofrii* DBVPG 5303 (Figure 3A and Figure 3D, respectively). In contrast, based on the numbers of sterilized coupons (Table 1; column six), the descending order for UV resistance was *A. fumigatus* ISSFT-021-30, *N. onofrii* DBVPG 5303, *B. pumilus* SAFR-032, and *G. stearothermophilus* ATCC 12980 (Table 1, columns 3–5).

It is often useful to examine log10 data as untransformed means to interpret changes in the actual numbers measured in the assays. To this end, the data in Figure 3 are summarized in Table 1 using the original untransformed numbers in the assays. Table 1, column 2 gives the overall values for spore or cell densities for all species on a ‘per coupon’ basis. The data in column 2 were then multiplied by the number of coupons tested for all assays in Figure 3 (i.e., 64 coupons exposed to UV were tested for each species) to yield the total number of all spores or cells exposed to the 1-sol LMO simulations. Data are ranked top-to-bottom, from the highest total spore densities per coupon (i.e., *B. pumilus* SAFR-032) to the lowest (i.e., *N. onofrii* DBVPG 5303). In all cases, greater than 99.99% of all spores or cells deposited on the coupons were killed by the 1-sol LMO simulations (Table 1, column 5), and between 50.0% (*A. fumigatus* ISSFT-021-30) and 84.4% (*G. stearothermophilus* ATCC 12980) of coupons were sterilized (Table 1, column 6).

SEM images collected during Exp-1 revealed that the vast majority of the monolayers were composed of single layers of spores or cells (Figure S3). However, in a few instances, multi-layered aggregates were observed in SEM images for *A. fumigatus* ISSFT-021-30, *B. pumilus* SAFR-032, and *G. stearothermophilus* ATCC 12980, but not for *N. onofrii* DBVPG 5303 (Figure S4, white boxes). Thus, the low numbers of surviving spores or cells reported in Figure 3 and Table 1 likely reflect outliers on coupons that were shielded by overlying spores or cells.

### 3.2. Experiment 2: Can the LMO UV Flux Sterilize Surfaces?

Based on the results in Exp-1, in which only a few outliers (i.e., survivors) were detected for all four species during 1-sol simulations, longer time steps were tested to determine if the LMO conditions could sterilize coupons. The UV-tolerant fungus, *Aspergillus fumigatus* ISSFT-021-30, and bacterium, *Bacillus pumilus* SAFR-032, were selected based on their known UV resistance [11,15] and the results from Figure 3 and Table 1. UV exposure times between 1 h and 12 sols (96 h of UV irradiation) were tested for both species at LMO conditions of ≤5 × 10^−5^ mbar and 37 °C. However, a new, more sensitive assay for survivors was developed to capture all viable spores per replicate per species and to determine the time steps all replicates would be killed by the LMO simulations.

The standard- and extended-MPN assays used in Exp-1 are noted for accurately determining the biocidal effects of UV irradiation down to the extinction points in which a few single-digit outliers dominate the survival plots (see Schuerger [18] and Schuerger and Headrick [19]). However, the MPN assays did not sample 100% of all extraction fluids, and thus, a few viable spores might have been missed. Thus, the new assay was designed to incubate replicates in separate 50 cc tubes with the appropriate liquid medium for each species. If a single spore survived on any given experimental replicate, the liquid media in the 50 cc conical tubes would become turbid, indicating microbial growth. For Exp-2, single coupons were used for individual replicates for *B. pumilus* SAFR-032 (*n* = 32), but three coupons were pooled to create individual replicates for *A. fumigatus* ISSFT-021-30 (*n* = 24). Thus, spore densities at T0 for both species were approx. 1.0 × 10^6^ spores per replicate.

**Figure 4:**
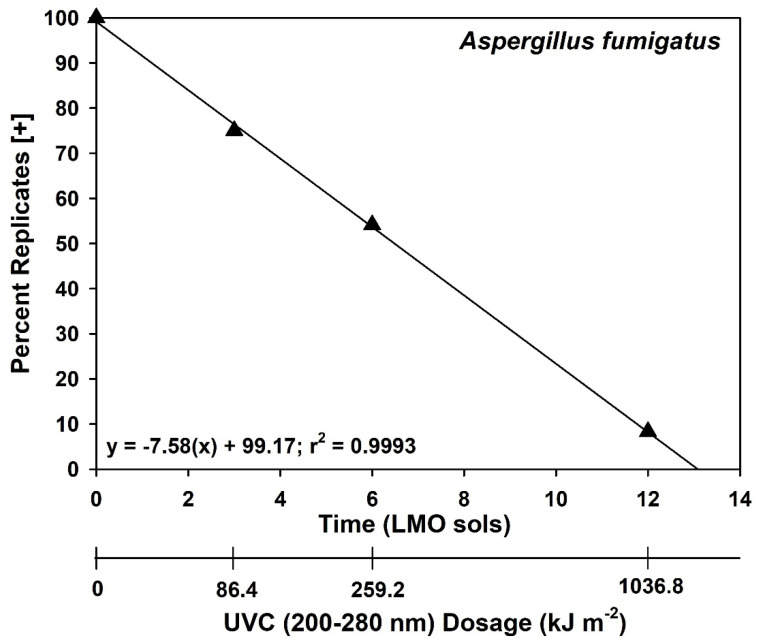
Survival of *Aspergillus fumigatus* ISSFT-021-30 spores during Exp-2 in which spores were exposed to low-Mars-orbit (LMO) conditions for up to 12 sols. Survivors (i.e., growth in potato dextrose broth (PDB) cultures) were observed in all LMO simulations with *A. fumigatus* ISSFT-021-30. However, a positive response in PDB could be induced by the survival of a single viable spore (*n* = 24 per treatment).

**Figure 5:**
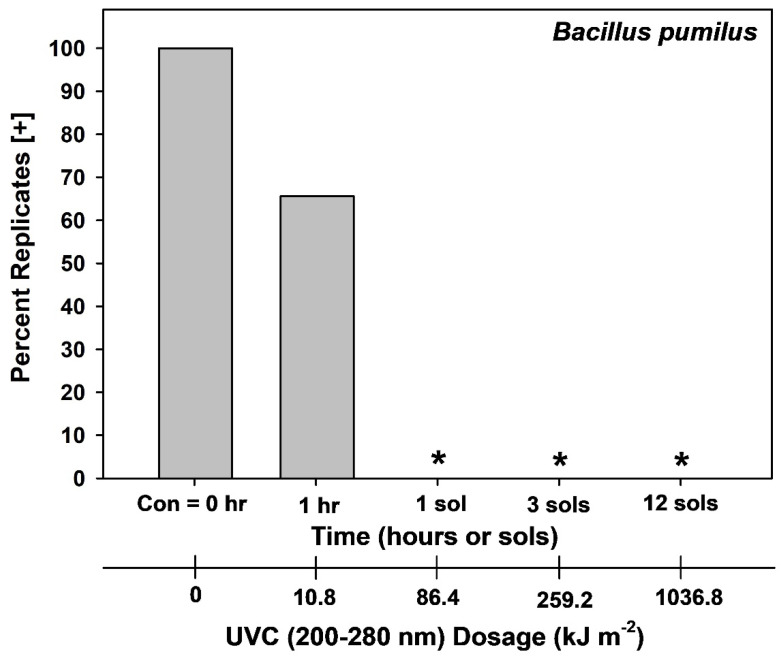
Survival of *Bacillus pumilus* SAFR-032 spores during Exp-2 in which spores were exposed to low-Mars-orbit (LMO) conditions for up to 12 sols. Thirty-five percent of doped *B. pumilus* SAFR-032 coupons exposed to LMO condition for only 1 h were sterilized (i.e., TSB cultures did not exhibit growth). In contrast, 100% of coupons, as denoted by an asterisk, (*n* = 32 per treatment) were sterilized when exposed to LMO conditions for 1, 3, or 12 sols.

Long-term LMO assays with *A. fumigatus* ISSFT-021-30 showed that a small number of viable spores persisted on some replicates after 12-sols of simulation (Figure 4), with 10% of replicates exhibiting detectable growth. In contrast, and divergent from Exp-1, replicates for *B. pumilus* SAFR-032 exhibited full sterility after 1-sol LMO simulations (Figure 5). In fact, approx. 40% of *B. pumilus* SAFR-032 replicates were sterilized after only 1 h of UV irradiation under LMO conditions. The likely explanation for the differences between Exp-1 (Figure 3) and Exp-2 (Figure 5) for *B. pumilus* SAFR-032 is the stacking of a few spores on coupon surfaces.

### 3.3. Experiment 3: UVC Attenuation by MMS Filters Versus Microbial Survival

**Figure 6:**
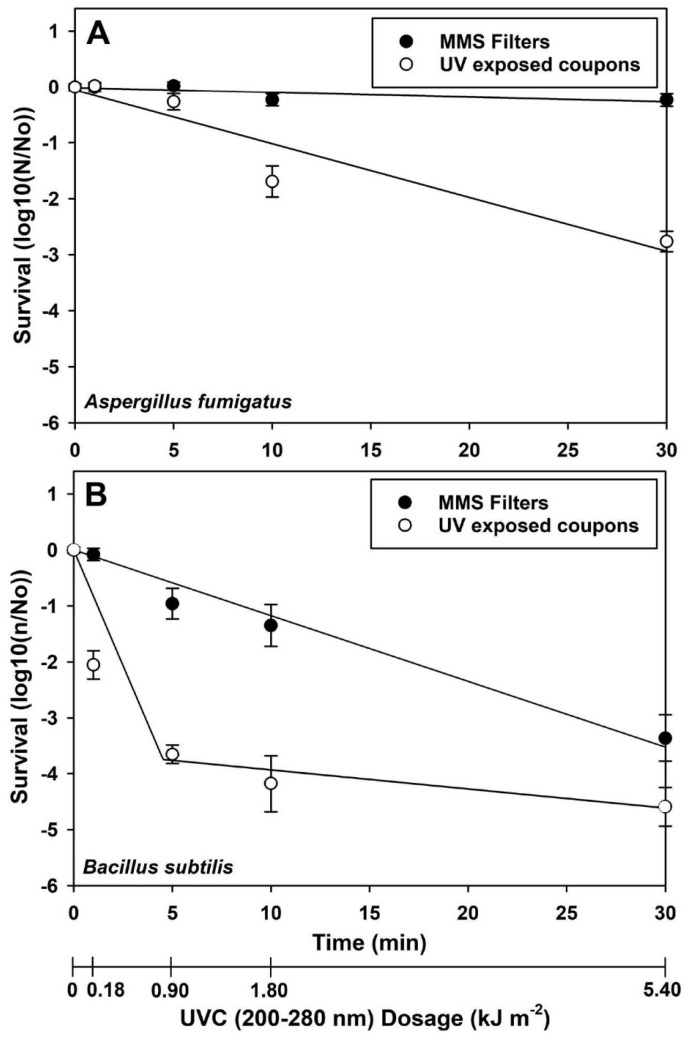
Survival of spores for *Aspergillus fumigatus* ISSFT-021-30 and *Bacillus pumilus* SAFR-032 during Exp-3 in which spores were exposed to low-Mars-orbit (LMO) conditions with UV irradiation between 0 and 30 min. Slope values and spline points (when appropriate) are given in Table 2 for the linear regression models. (**A**) Slope values for the MMS filters and UV-exposed coupons were significant (*n* = 16; *p* ≤ 0.01) for *A. fumigatus* ISSFT-021-30, indicating that UV irradiation was inactivating spores under LMO conditions. (**B**) The slopes for the UV-inactivation of *B. pumilus* SAFR-032 spores were significantly more negative than *A. fumigatus* ISSFT-021-30 (Table 2), indicating a lower UV-resistance for *B. pumilus* SAFR-032 spores. The spline point for the *B. pumilus* SAFR-032 1st and 2nd phases of biocidal activity for UV-exposed spores was observed at x = 4.88 min and y = −3.811 log reductions. Error bars are 95% CI ranges for treatment means.

Exp-3 was designed to determine the linear models for biocidal UV effects under LMO conditions between 1 and 30 min, with and without shielding from Mars particles. MMS filters in fused-silica glass were used as a proxy for Mars particles up to 20 µm, a thickness that represents a rare but credible size for aerosolized or wind-borne particulate on Mars [9]. Results could be used to model the direct biocidal inactivation kinetics of solar UV simulations under LMO conditions but shielded by thin layers of Martian regolith. First, the inactivation of *A. fumigatus* ISSFT-021-30 spores followed linear models for both the UV-exposed and MMS-shielded spores (Figure 6A; Table 2). The MMS filters were expected to allow approx. 5–10% transmittance of the UVC spectrum from the xenon arc UV beam (Figure 2). Thus, the slope value for the MMS shielded spores for *A. fumigatus* ISSFT-021-30 was significant (*p* < 0.0001) but with a shallow negative slope. Overall, the UV-induced inactivation rate of *A. fumigatus* ISSFT-021-30 spores was 10.7-times faster without a MMS filter, consistent with a >90% reduction in UVC flux (Figure 6A and Table 2).

Inactivation kinetics for *B. pumilus* SAFR-032 spores were significantly faster (i.e., steeper slopes) than *A. fumigatus* ISSFT-021-30, reaching lower inactivation levels close to −4 and −5 logs after 30 min (Figure 6B; Table 2). Interestingly, the UV-exposed layers of *B. pumilus* SAFR-032 spores exhibited a bi-phasic response in which a very steep kill curve was observed for the 1st phases between 0 and 5 min, resulting in over −4 logs of bioburden reduction. The steep 1st phase likely represents the inherent biocidal effects of UVC irradiation on spore survival, and the shallower 2nd phase likely represents the survival kinetics of outliers shielded by overlying spores or by capture within pits and crevices [18,19]. Although only the first phase was observed with the MMS filter in place, it showed a similar effect to that observed with *A. fumigatus* ISSFT-021-30, and was 6.7-times faster without the MMS filter, consistent with a >90% reduction in UVC flux.

### 3.4. Experiment 4: High-Resolution Scans of Spore Layers and UV Transmittance of Stacked Spores

As results emerged from Exp-1 to Exp-3, our team decided to examine the 3D structure of spore monolayers more closely with two goals in mind: (1) to characterize the nature of spore aggregates on coupons, and (2) to determine the UV-transmittance through multi-spore stacks on the coupons. Two instruments were ideal for the examinations; namely, the Keyence VK-X3100 Laser Scanning Confocal Microscope and the CRAIC 2030PV PRO Microspectrophotometer, respectively. Monolayers of the model species *A. fumigatus* ISSFT-021-30 on aluminum coupons were created in the Florida PAC lab, UV-irradiated for 6 h with the PAC UV-VIS-NIR xenon arc beam, and shipped to JPL.

**Figure 7:**
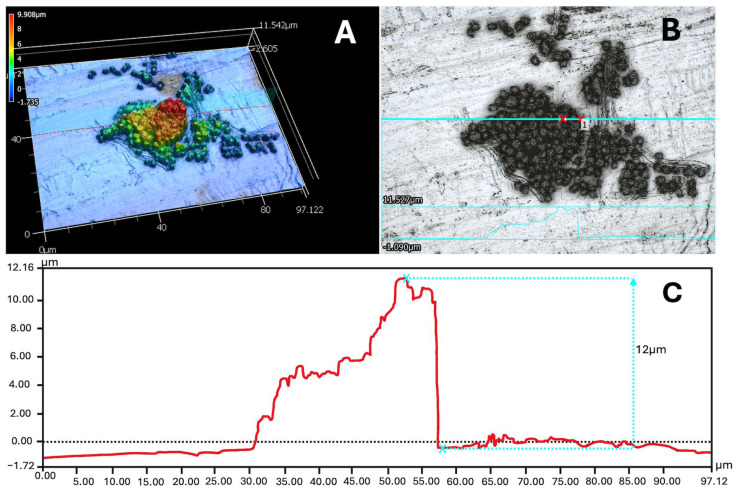
Laser Scanning Confocal Microscope imagery of *Aspergillus fumigatus* ISSFT-021-30 spores on aluminum coupons post-UV exposure. (**A**) Three-dimensional topographic scan of an *A. fumigatus* ISSFT-021-30 cell cluster. (**B**) Two-dimensional image of the same cell cluster. The blue line #1 across the center of the image represents the cross-section of cell cluster height pictured in panel (**C**). (**C**) Profile of transect across a cell cluster pictured in panels (**A**,**B**). Max height of cluster was approximately 12 μm.

First, the 3D structures of several spore stacks were characterized, in which the 3D structure (e.g., Figure 7A) was mapped against a 2D light image (Figure 7B). The 3D imaging identified that *A. fumigatus* ISSFT-021-30 spore stacks could form on coupons up to 12 µm thick (Figure 7C) and represented approx. six spore layers. The blue transect line in Figure 7B shows the 3D topology of a spore stack that is given in Figure 7C. However, the observed stacking was not common on aluminum coupons. In most cases, monolayers were composed of single spore layers spread randomly (Figures S2 and S3) on the 1 cm × 1.5 cm wide visible dried spore monolayers on aluminum coupons (Figure S1).

**Figure 8:**
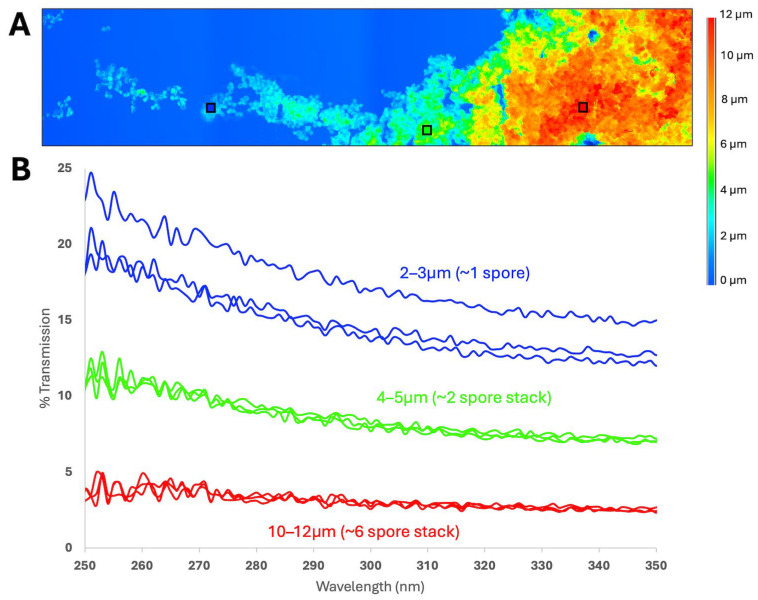
Ultraviolet (UV) transmission measurements of non-irradiated *A. fumigatus* ISSFT-021-30 cells deposited on a UV fused-silica glass slide. (**A**) Heatmap of cell depth across a section of the cell deposit. Colored squares mark areas of various cell depths where UV transmission was measured. (**B**) UV transmission through various cell deposits corresponding to different cell stack values. As the cell stack depths increased, UV transmission decreased. However, even when UV was measured through 10–12 µm of cells (approx. 6 spore layers), UV transmittance was >0.

Next, measurements of UV transmissions through various cell stacks of *A. fumigatus* ISSFT-021 showed that single layers of conidia could attenuate more than 80% of UVC at 250–280 nm and a cell stack approximately the height of six spore layers can attenuate more than 95% of UVC (Figure 8). However, in no case did we observe 100% of UVC photons attenuated by any of the spore stacks measured. Figure 8A depicts the 3D heat map of a spore cluster on a single coupon. Figure 8B shows three independent scans of three spore layers, corresponding to stacks approx. 1, 2, or 6 spores tall. Although spore stacking and UV transmittance through multi-layers of spores were only measured for *A. fumigatus* ISSFT-021-30 using the CRAIC and LSCM instruments, spore stacking was also observed in standard SEM imaging for *B. subtilis* and *G. stearothermophilus* ATCC 12980, but not for *N. onofrii* DBVPG 5303 (Figure S3).

## 4. Discussion

The MSRP campaign includes three spacecraft (i.e., Perseverance, the SRL + MAV joint lander, and the ERO), working together to return Martian regolith, dust, and rock samples to Earth. Currently, the Perseverance rover is collecting the samples, placing them in sealed sample tubes, and depositing the tubes on the surface in at least two caches. The entire campaign is likely to span 1 to 2 decades, during which time Earth outbound microbial bioburden (i.e., henceforth called Earth-sourced microorganisms) will be inactivated over time by several space and Mars biocidal factors (e.g., solar heating, solar UV, desiccation, vacuum [7,8,14]).

There are two sources of microbial contamination of concern during the MSRP missions. First, Earth-sourced microbes could remain viable during the outbound trip, Mars surface activities, and the return inbound trip, leading to false positives for the detection of an extant Martian microbiota. Second, if an extant Mars microbiota does exist, then Mars-sourced microorganisms picked up during sample collection would be a potential back-contamination hazard for Earth. The research described here was designed to develop inactivation rates of four UV- and heat-resistant microbial species to examine the effects of LMO conditions on surviving Earth-sourced microbiota and to develop a 1st-order model on how a plausible Mars-sourced microbiota might survive on external surfaces of spacecraft, such as the OS or ERO, in LMO. The primary goal of the current study was to examine the effects of LMO-simulated conditions of solar UV irradiation, high-vacuum, and temperature on four UV-resistant microorganisms as proxies for predicting the survival of out-bound spacecraft bioburden and any in-bound Mars microbiota.

### 4.1. Biocidal Nature of the LMO Environment for Earth-Sourced Microorganisms

Cells or spores (henceforth called spores) of four microbial species were exposed to 1-sol LMO simulations to characterize the effects of UV irradiation, vacuum, and temperature on microbial survival. The four species were selected based on recent studies that proposed UV- and heat-resistant model microbial species for planetary protection experiments [11,15].

Of the three environmental conditions tested, UV irradiation was the dominant biocidal factor in the LMO simulations, with vacuum and heat effects found to be of no or minor importance in the inactivation of the microbial spores. The 1-sol LMO exposures to UV irradiation killed almost all spores in the assays. Results match several recent studies on the biocidal effects on spacecraft microorganisms caused by Mars surface conditions [16,17,30,31,32,33], lunar surface environmental factors [14], and interplanetary space [8,34]. In all cases, inactivation levels (Figure 3) were very close to the average bioburden applied to coupons (Table 1, column 2).

However, spore inactivation rates for all species under LMO conditions in Figure 3 appear to suggest that *N. onofrii* DBVPG 5303 is the most UV-resistant species because bioburden reductions achieved only −4 logs while bioburden were reduced approx. −5 logs for *A. fumigatus* ISSFT-021-30 and approximately −6 logs for *B. pumilus* SAFR-032 and *G. stearothermophilus* ATCC 12980. This effect is an artifact of the divergent starting populations of the four species tested because spores were not identical in size. The larger the spore diameters, the lower the numbers of spores that could be applied to coupons while limiting spore stacking. For example, the smallest spores from *B. pumilus* SAFR-032 (~1 µm) and *G. stearothermophilus* ATCC 12980 (~1.25 µm) allowed high numbers of spores to be applied to individual coupons without excessive stacking. In contrast, the spores of *A. fumigatus* ISSFT-021-30 (~2–3 µm) and *N. onofrii* DBVPG 5303 (~4.5 µm) were significantly larger, requiring lower spore densities per coupon to avoid spore stacking. Thus, spore crowding was a key driver in determining the apparent levels of UV inactivation that could be resolved by the protocols used herein.

Another way to look at microbial survival rates of the four species exposed to LMO conditions is to present the inactivation data as rational numbers (i.e., integers) and not logs. Table 1 presents the inactivation data in two ways. First, column 3 (i.e., from the left) gives the total number of spores applied to all 64 coupons (i.e., replicates) for each species. Column 4 gives the total number of spores recovered by the described MPN protocols for all 64 coupons per species. And column 5 gives the percent of dead spores for all 64 coupons per species. For example, 1.20 × 10^8^ spores of *B. pumilus* SAFR-032 (i.e., 120 million spores) were applied to all 64 coupons in Exp-1, and only 100 spores were recovered by the MPN protocol. Thus, the vast majority of spores (i.e., 120 million spores minus 100 spores = 119,999,900 spores killed) yielded >99.9999% of spores were killed by the 1-sol LMO simulations. Second, Table 1 also gives the percentage of coupons sterilized (def., no viable spores recovered per coupon) in the 64 coupons assayed per species. In the cases of *A. fumigatus* ISSFT-021-30 and *B. pumilus* SAFR-032, the numbers of coupons sterilized by the 1-sol simulations were 50 and 79.7%, respectively.

Taken together, the data in Figure 3 and Table 1 make a strong case that LMO conditions will likely be effective in sterilizing most sun-exposed spacecraft surfaces because the high inactivation rates presented here for Exp-1 are only for 1-sol simulations. If the external OS or ERO surfaces will remain in LMO for up to 30–70 sols [1], the kill rates given here will be repeated up to 30–70 times (depending on hardware spin state and pointing relative to the sun) during the course of the OS and ERO rendezvous, docking, OS capture, and ERO launch back to Earth. It is useful to think of microbial inactivation rates on both a log and integer basis in order to understand how significantly biocidal the LMO conditions are to spacecraft bioburden.

A key finding here was observed when UV-transmittance rates were estimated for multi-layered spore aggregates of *A. fumigatus* ISSFT-021-30. Results indicated that UVC photons were able to penetrate up to 10–12 µm thick layers of *A. fumigatus* ISSFT-021-30 spores composed of 5–6 layers of spores, but the dose and reduction rates would be lower than for fully exposed spores.

### 4.2. Estimating SAL Values for Earth-Sourced Microorganisms on Spacecraft in LMO

In order to identify the SAL values for two species (i.e., −12 log reductions [10,11]), the 12-sol experiments were designed to determine at what LMO time steps zero survivors would be observed for all replicates in the UV-resistant species *A. fumigatus* ISSFT-021-30 and *B. pumilus* SAFR-032 (Exp-2). In the case of *A. fumigatus* ISSFT-021-30, approx. 10% of coupons at 12-sols retained at least one viable spore (Figure 4). In contrast, 100% of *B. pumilus* SAFR-032 coupons were sterilized for 1-, 3-, and 12-sol LMO simulations (Figure 5). Results for *A. fumigatus* ISSFT-021-30 were not surprising because spores of the fungus have thick walls and contain melanin, both factors can increase survival rates of microorganisms against UV irradiation (e.g., Onoda et al., [35]; Pacelli et al., [36]). Based on the data for *A. fumigatus* ISSFT-021-30 in Figure 3 and Table 1, the vast majority of spores for *A. fumigatus* ISSFT-021-30 were inactivated during 1-sol LMO simulations. The data are arguably similar for *A. fumigatus* ISSFT-021-30 among Figure 3 and Figure 4, and Table 1, but likely differ based on the shading effects of multiple stacks of spores on coupons. Results for *B. pumilus* SAFR-032 suggest that biocidal effects of the LMO simulations were slightly faster in Exp-2 (Figure 5) compared to Exp-1 (Figure 3). We interpret the subtle results discussed here as examples of experimental error in the LMO assays, likely related to spore/cell stacking.

Based on the data in Figure 3 and Figure 4, and in Table 1, we estimate that achieving one SAL for *A. fumigatus* ISSFT-021-30 might require longer than 12 sols in LMO, and most likely might extend to 20–24 sols. In contrast, a SAL of 2 sols for *B. pumilus* SAFR-032 might be derived by doubling the 100% inactivation rate of 1 sol given in Figure 5 (i.e., doubling the −6 log biocidal effect in Figure 5 to −12 logs of bioburden reduction for SAL; sensu Sandle [10] and Craven et al. [11]). Thus, if the OS remains in LMO for 30–70 sols, the external surfaces might exceed a SAL treatment by 2.9- to 3.5-fold for Earth-sourced microorganisms if we use *A. fumigatus* ISSFT-021-30 as the single modeled species, or by over 15-fold if the results for *B. pumilus* SAFR-032 are used.

### 4.3. Dust May Lower Bioburden Inactivation Rates by UV Irradiation

The discussion in Section 4.1 is based on the premise that microbial spores are not covered by UV-attenuating Martian dust particles. Furthermore, in certain architectures (e.g., [1]) or anomalous scenarios, it is expected that some airborne Mars dust may adhere to the OS and MAV during surface operations, and thus, dust may play a role in absorbing UV irradiation during LMO operations with the OS or ERO spacecraft. Experiment 3 was designed to characterize the effects of dust reducing incident UV irradiance on the survival of two UV-resistant microbial species. Absorption by organic contaminants was not tested directly, but the effects are likely included in the experiments because organics are abundant during cultivation and added to promote monolayer deposition during drying. Thus, non-spore and non-cell organic material would be expected to dwarf the ≤0.1% found in PHMM (e.g., the Stern et al. study of organics detected on Mars [37]).

The plots with MMS filters in place for *A. fumigatus* ISSFT-021-30 (Figure 6A) and *B. pumilus* SAFR-032 (Figure 6B) indicated much shallower slopes for the inactivation kinetics for spores covered by the MMS filters. In particular, the plot for the MMS filter with *A. fumigatus* ISSFT-021-30 spores (Figure 6A) appears nearly horizontal. However, considering the linear models and slope *p* values in Table 2, the *p* values confirm that the slope for *A. fumigatus* ISSFT-021-30 covered by the MMS filters had a significant negative slope (*p* < 0.0001), indicating that spore death at 30 min was significantly greater when compared to 0 min. Using the linear models in Table 2, inserting a log reduction of −12 logs for [y], and solving for time [x], we estimate that SAL values for *A. fumigatus* ISSFT-021-30 exposed directly to LMO UVC fluence rates or covered by dust would be approximately 2 or 22 h, respectively.

Thus, the MMS filters slowed UV-specific microbial reduction and would extend the exposures required to establish one SAL, but not by months or years. We conclude that even if UV attenuation by dust protecting viable spores from direct sunlight occurs on spacecraft in LMO, the reduced biocidal processes would be extended slightly, and the resulting SAL values remain reasonable for flight durations. Extrapolating from the 2nd phase rate (Figure 6B), *B. pumilus* SAFR-032 would require a total exposure of 4.2 h to reach one SAL. Given that the reduction rate with MMS-filtered light was 14.8% of the unfiltered biocidal rate, this would correspond to a SAL of 28.5 h of direct sun exposure in LMO for *B. pumilus* SAFR-032 spores protected by ≤20 µm of Mars dust.

The SAL values above may be conservative estimates for two reasons. First, the MMS filters degraded over time during Exp-3. Data in Figure 2B demonstrated that the UV scans from 200 to 400 nm—before and after the MMS filters were used in Exp-3—showed a 50% lower value at 200 nm after Exp-3 was completed. The UVC attenuation was much lower at higher wavelengths; for example, attenuation was only 9% at 400 nm. The loss of transmittance through the MMS filters was most likely due to the aging effects of the RTV matrix during the 130 °C sterilization cycles and the UVC exposures themselves. Increased attenuation of UVC photons over time by dust would not be expected on actual spacecraft surfaces in LMO because the dust-holding matrix RTV would be absent. Second, Schuerger et al. [17] demonstrated that UVC photons can scatter around, reflect from surface defects, or penetrate dust particles on proxy spacecraft surfaces, rendering it possible that fine dust particles on the OS or ERO might not be capable of attenuating all biocidal UVC irradiation. If both mechanisms are active, the SAL values estimated above for dusty conditions might be more aligned with the UV-exposed values than with the MMS filter values. If confirmed, the MMS filters give us a reasonable estimate of SAL of ≤3.6 sols for surfaces exposed directly to the sun on a rotating body (a particle or on hardware) in LMO, but more work is required to partition the role of RTV in the assays presented here.

The single factor that might greatly extend SAL estimates for model species in LMO is the process of stacked spores on spacecraft surfaces. Here, we demonstrate that spore stacking occurs on the periphery of spore monolayers on the experimental aluminum coupons (Figure 7), and thus, survival might have been enhanced in all of the tests presented above if stacking were a dominant factor. In contrast, reviewing the literature on microbial bioburden assays of spacecraft surfaces prior to launch clearly indicates that microbial cells are recovered at extremely low densities (e.g., Cooper et al., [38]), and thus, significant spore stacking might not occur on actual spacecraft surfaces. Spore stacking might be an artifact of the approach used here, of placing 2 × 10^6^ spores on approximately 1 cm^2^ of coupon surface.

## 5. Conclusions

Solar UVC is a key lethality factor for organisms exposed to the space flight environment. The results presented here build off previous work on the lethality that microbial contaminants might experience on space flight hardware during the outbound phases of interplanetary missions and from surface operations [14,39] and extend it to the potentially shorter durations of exposure that some hardware might experience during a sample return mission from Mars. The mission concept developed for the MSR Campaign would result in all returning hardware being exposed to solar UVC in LMO for multiple sols, at a minimum, and much of that hardware would be expected to be in-flight for ≥5 years.

Results support the following three overarching conclusions: First, as little as 1-sol exposures to UVC, high-vacuum, and plausible surface temperatures on a spacecraft in LMO reduced even UV-resistant microorganisms by multiple orders of magnitude (Figure 3) and were sufficient enough to kill >99.99% of spores/cells from four species (Table 1). Second, stacked spores of *A. fumigatus* ISSFT-021-30 to depths of 10–12 µm could not fully prevent UV transmission (Figure 7 and Figure 8), but did extend predicted SAL levels to longer time steps than 1 sol. And third, the UV attenuation observed by stacked spores of *A. fumigatus* ISSFT-021-30 offers an explanation for why the biocidal effects among several different experiments diverged slightly. We argue here that the ‘experimental error’ associated with stacked spores/cells is a primary cause of divergent results in Figure 3, Figure 4, Figure 5 and Figure 6.

In summary, this work demonstrates that organisms associated with surfaces and particulate matter, as could be expected during the MSRP Campaign, can be inactivated by solar UVC in LMO. This work also demonstrates clearly that understanding solar UVC lethality in space and, importantly, during testing, requires defining and understanding the worst-case microbial targets in terms of how they associate with biological or particulate matter and how those associations may reduce the biocidal effects of UVC irradiation. In this study, 20 µm of Mars simulant transmitted a solar UV dose rate about 5–10% of unfiltered illumination (Figure 2) and the maximum observed spore stacking resulted in about 4–5% UVC transmission (Figure 8B). Together, these parameters might require significantly more time to achieve the same results with a worst-case organism relative to a fully exposed organism (with the caveat about how spore stacking affected the results given above). Critically, only a fraction of organisms would be so protected, as evidenced by the rapid 4-log reduction in even *A. fumigatus* ISSFT-021-30 populations. The worst-case combination would only be relevant if it occurs on the target hardware with a probability at or above the required SAL value. Overall, with conservative estimates of microbial aggregate, inorganic particle size distributions, and their UVC transmission rates, UVC lethality is likely sufficient to mitigate biological contamination in LMO for the MSR Campaign and similar interplanetary missions.

## Figures and Tables

**Table 1:** Spore survival for four species of UV-resistant microorganisms on Al-coupons exposed to 1-sol LMO simulations (estimated from Exp-1 data; see Figure 3).

Species	T = 0 Spores per Coupon	Total # of Spores on 64 Coupons	Post-UV Survival (# of Spores)	Percent (%) Dead Spores	Percent (%) Sterilized Coupons
*B. pumilus* SAFR-032	1.88 × 10^6^	1.20 × 10^8^	100	>99.9999	79.7
*G. stearothermophilus* ATCC 12980	9.15 × 10^5^	5.86 × 10^7^	80	>99.9999	84.4
*A. fumigatus* ISSFT-021-30	1.67 × 10^5^	1.07 × 10^7^	625	99.994	50.0
*N. onofrii* DBVPG 5303	2.38 × 10^4^	1.52 × 10^6^	130	99.991	71.9

**Table 2:** Linear models for data in Figure 6.

Species	UV vs. MMS	Linear Models ^1^	Slope *p* Values	r^2^
*Aspergillus fumigatus* ISSFT-021-30				
	MMS filters	y = −0.0092(x)	<0.0001	0.373
	UV exposed coupons	y = −0.0986(x)	<0.0001	0.905
*Bacillus pumilus* SAFR-032				
	MMS filters	y = −0.1162(x)	<0.0001	0.901
	UV; 1st phase	y = −0.7816(x)	<0.0001	0.889
	UV; 2nd phase	y = −0.0328(x) − 3.651	0.0010	0.211
	spline	x = 4.88, y = −3.81		

^1^ Linear models were determined with PROC REG in SAS; see main text.

## Data Availability

Raw data for Figure 3 are given as separate pages in Table S1. Data for Figure 4 and Figure 5 are given in Table S2. Data for Figure 6 are given in Table S3. Data for Figure 8B are given in Table S4. Note that the instrument output for Figure 7C is not available in a tabular form.

## Supplementary Materials

The following supporting information can be downloaded at: https://www.mdpi.com/article/10.3390/microorganisms14051158/s1, Figure S1: Spore and cell narrow ‘coffee rings’ on the perimeters of the ~20 mm × 15 mm dimensional square spots on aluminum coupons.; Figure S2: Keyence (model VHX-7000) high-resolution video images of spore monolayers on aluminum coupons for *Aspergillus fumigatus* ISSFT-021-30, *Bacillus pumilus* SAFR-032, *Geobacillus stearothermophilus* ATCC 12980, and *Naganishia onofrii* DBVPG-5303.; Figure S3: SEM images of spore monolayers on aluminum coupons for *Aspergillus fumigatus* ISSFT-021-30, *Bacillus pumilus* SAFR-032, *Geobacillus stearothermophilus* ATCC 12980, and *Naganishia onofrii* DBVPG-5303.; Figure S4: SEM images of multi-layers of spores (white boxes) were observed along the borders (i.e., coffee rings) of the doped surfaces for three species of the UV-resistant microorganisms exposed to low-Mars-orbit (LMO) conditions in the PAC.

## Abbreviations

ATCC	American Type Culture Collection
AU	astronomical unit
CCRS	Capture Containment and Return System
CMC	critical micelle concentration
ERO	Earth Return Orbiter
ESA	European Space Agency
JPL	Jet Propulsion Lab
LMO	low-Mars-orbit
LN2	liquid nitrogen
*Ls*	solar longitude
LSCM	Laser Scanning Confocal Microscope
MAV	Mars Ascent Vehicle
mbar	millibar
MMS	Mojave Mars simulant
MPN	Most Probable Number
MSR	Mars Sample Return
MSRP	Mars Sample Return Program
NASA	National Aeronautical and Space Administration
NIR	near-infrared
NS	not significant
OD	optical density
OS	Orbital Sample holder in the MSR architecture
PAC	Planetary Atmospheric Chamber
PDA	potato dextrose agar
PDB	potato dextrose agar
PHMM	potentially harmful Mars material
PP	planetary protection
PVA	polyvinyl alcohol
RTV	room temperature vulcanized
SAL	sterility assurance level
SAS	Statistical Analysis System
SDIW	sterile deionized water
SEM	scanning electron microscopy
SRL	Sample Retrieval Lander
T	transmittance
TC	thermocouple
TSB	trypticase soy agar
UV	ultraviolet irradiation
UVC	ultraviolet light in range C (200–280 nm)
VIS	visible light (400–700 nm)
W	watts
