# Supplementary material for: Biocidal Conditions in Low-Mars-Orbit Can Inactivate Bioburden on External Mars Spacecraft Surfaces and Dust Particles Within a Few Sols
Source: Microorganisms. 2026 May 20;14(5):1158. doi: 10.3390/microorganisms14051158 (PMC13209720; doi:10.3390/microorganisms14051158)
Supplement: Supplementary file 1 [file microorganisms-14-01158-s001.zip › Schuerger et al_JPL Exps_supplemental_CL26_1557.pdf]

Supplementary Material

Andrew C. Schuerger [1], Petra Schwendner [1], Lisa Guan [2], Jerami Mennella [2], Nicholas Heinz [2], Ioannis Mikellides [2] and Brian G. Clement [2,*]

[1] Department of Plant Pathology, University of Florida, Gainesville, FL 32611, USA; schuerg@ufl.edu (A.C.S.); science.schwendner@outlook.com (P.S.)
[2] Jet Propulsion Laboratory, California Institute of Technology, Pasadena, CA 91109, USA; lisa.guan@jpl.nasa.gov (L.G.); jerami.mennella@jpl.nasa.gov (J.M.); nicholas.a.heinz@jpl.nasa.gov (N.H.); ioannis.g.mikellides@jpl.nasa.gov (I.M.)

* Correspondence: brian.g.clement@jpl.nasa.gov

Funding support to the University of Florida for the creation of this work was provided by the Jet Propulsion Laboratory, California Institute of Technology, under grant JPL-1694993. Funding support to the Jet Propulsion Laboratory, California Institute of Technology, was provided as part of the Mars Sample Return Program under a contract with the National Aeronautics and Space Administration (80NM0018D0004).

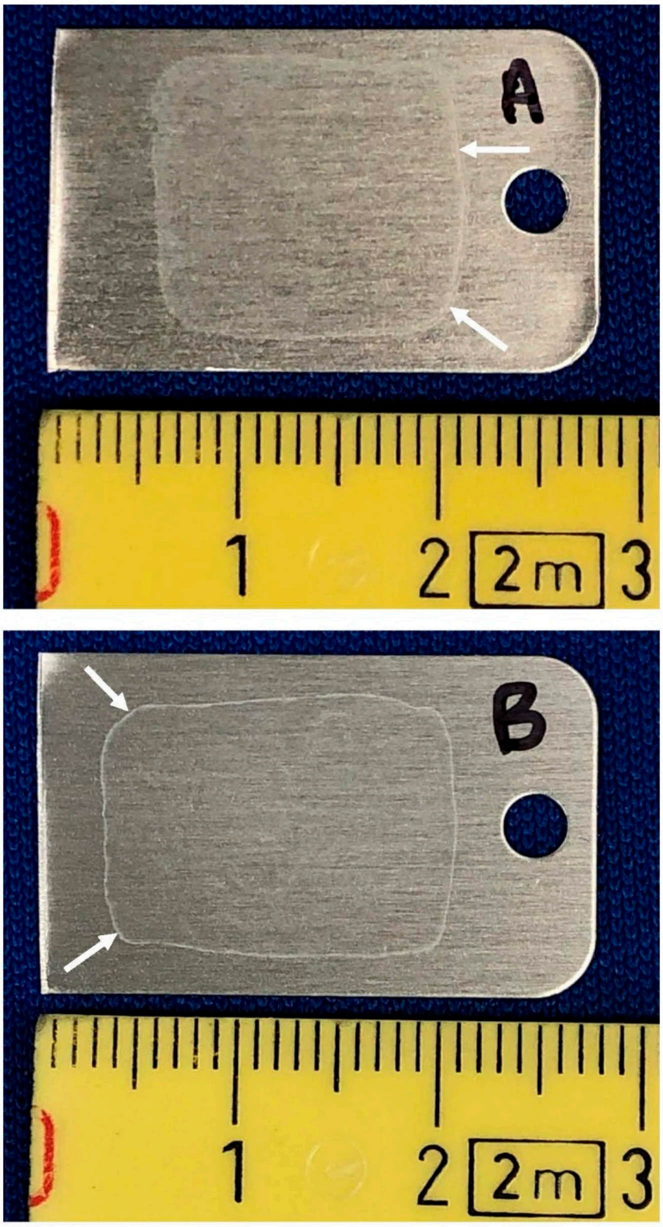

Figure S1. Spore and cell monolayers exhibited narrow 'coffee rings' (c.f., references 20 and 21 in the associated paper) on the perimeters of the ~20 x 15 mm dimensional square spots on aluminum coupons. Most stacking of spores or cells occurred in the coffee rings but were not uniformly stacked around the perimeters of the monolayer spots. Little to no stacking of spores or cells was observed on the interiors of the monolayers.

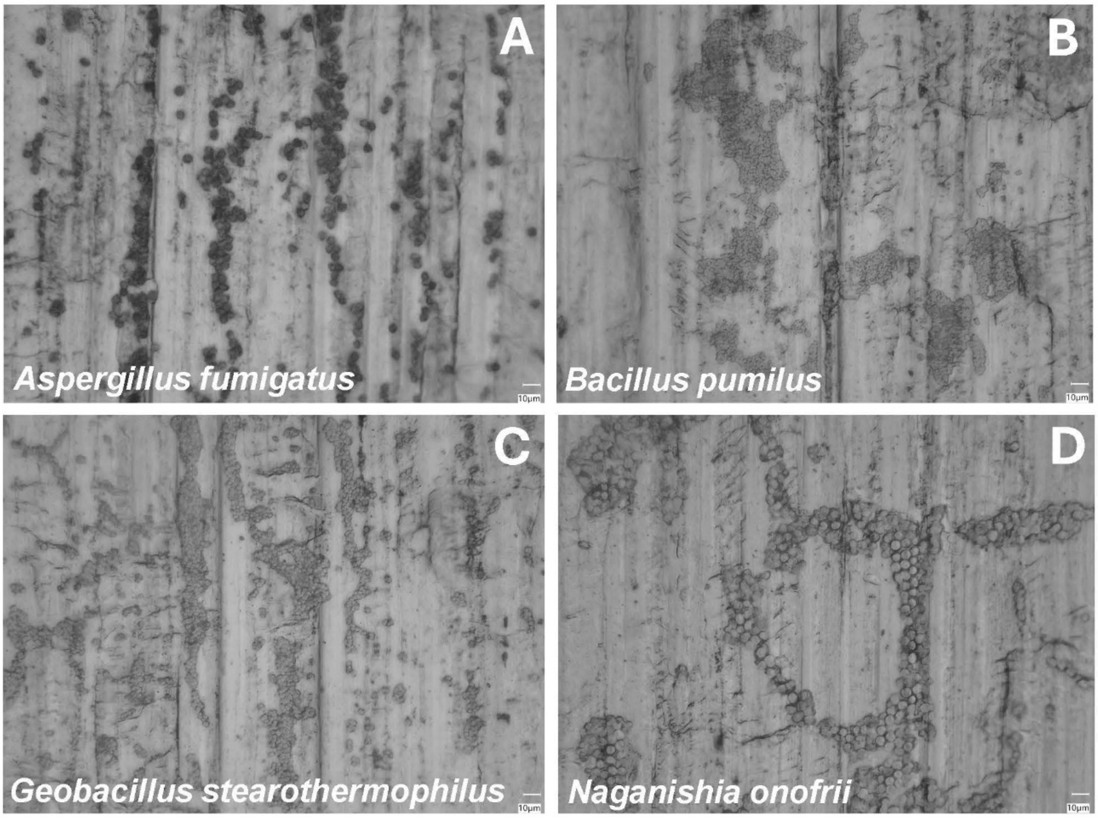

Figure S2. Keyence (model VHX-7000) high-resolution video images of spore monolayers on aluminum coupons for *Aspergillus fumigatus* ISSFT-021-30 (**A**), *Bacillus pumilus* SAFR-032 (**B**), *Geobacillus stearothermophilus* ATCC 12980 (**C**), and *Naganishia onofrii* DBVPG 5303 (**D**). Spores for each species were ~2-3 µm in diameter for *A. fumigatus* ISSFT-021, ~1 µm for *B. pumilus*, ~1.25 µm for *G. stearothermophilus*, and ~4.5 µm for *N. onofrii* DBVPG 5303. Mucilage deposits from cell cultures were observed only with monolayers of *N. onofrii* DBVPG 5303. Scale bars equal 10 µm.

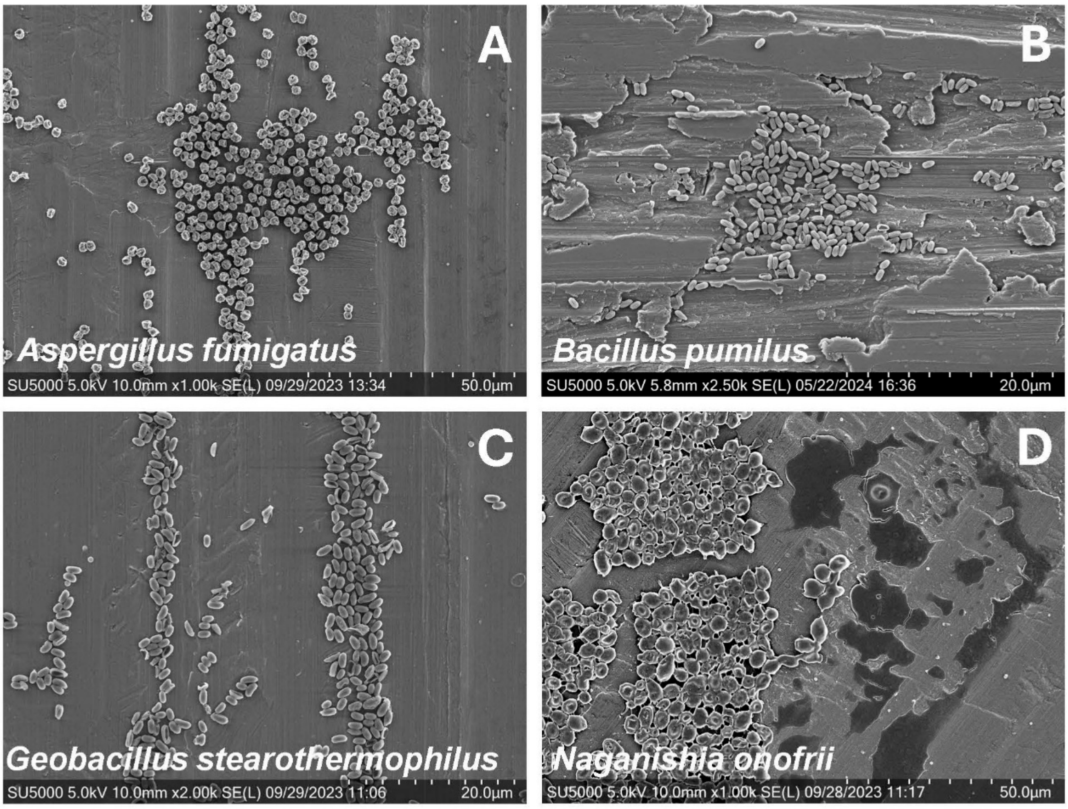

Figure S3. SEM images of spore monolayers on aluminum coupons for *Aspergillus fumigatus* ISSFT-021-30 (**A**), *Bacillus pumilus* SAFR-032 (**B**), *Geobacillus stearothermophilus* ATCC 12980 (**C**), and *Naganishia onofrii* DBVPG 5303 (**D**). Spore monolayers exhibited single-layers of spores or cells within ~20 x 15 mm spore deposits in which spore/cell mucilage was generally absent (i.e., for *A. fumigatus* ISSFT-021, *B. pumilus* SAFR-032, and *G. stearothermophilus* ATCC 12980). In contrast, *N. onofrii* DBVPG 5303 monolayers exhibited extensive spore mucilage that surrounded the vegetative cells. The coupons for Exp-1 and Exp-2 exhibited smooth backgrounds with very slight undulations (**Figs**. **S3A, C**, and **D**). However, a new population of aluminum coupons were purchased for Exp-3 in which the surfaces were noticeably rougher (**Fig. S3B**).

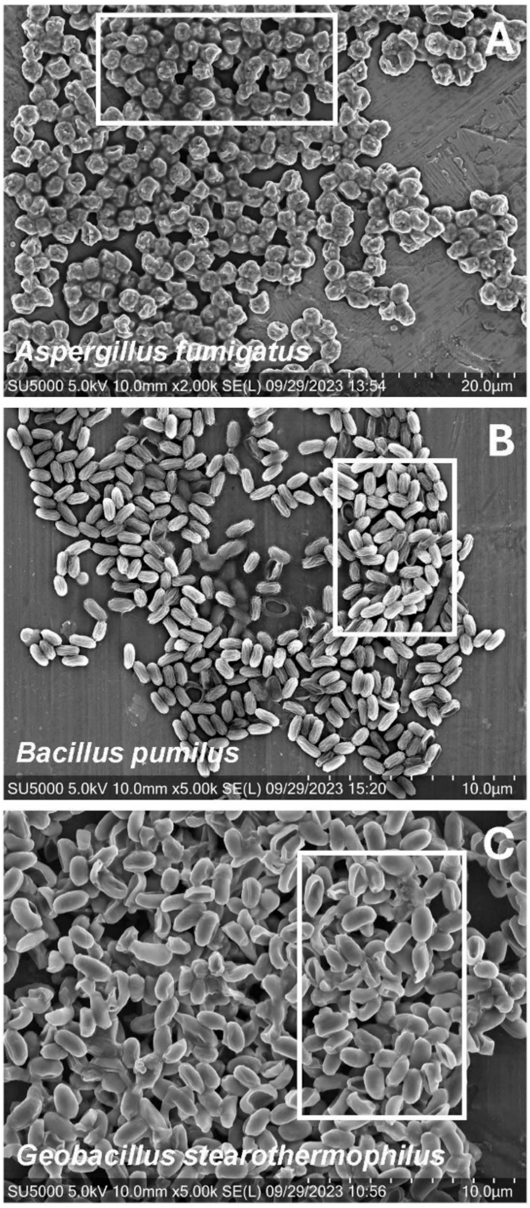

Figure S4. SEM images of multi-layers of spores (white boxes) were observed along the borders (i.e., coffee rings) of the doped surfaces for three species of the UV-resistant microorganisms exposed to low-Mars-orbit (LMO) conditions in the PAC. (**A**) Conidia of *Aspergillus fumigatus* ISSFT-021-30 averaged 2-3 µm in diameter and would stack into 2-6 layers along the coffee rings of the spore layers. (**B**) Endospores of *Bacillus pumilus* SAFR-032 averaged 1 µm for their long axis and would form 2-3 layered aggregates along the coffee rings of the applied spores. (**C**) Endospores of *Geobacillus stearothermophilus* ATCC 12980 averaged between 1.5 and 2 µm in length and would stack into multi-layers estimated to be between 3-5 spore layers thick. In contrast, multi-layers of spores for *Naganishia onofrii* DBVPG 5303 were not observed (not shown here). SEM magnifications are given in each legend adjacent to the letter ‘k’.
